# Two Cases of Iatrogenic Lumbar Foraminal Disc Herniations

**DOI:** 10.1155/2021/5546530

**Published:** 2021-12-24

**Authors:** Ryota Taniguchi, Osamu Kawano, Takeshi Maeda, Yasuharu Nakajima, Yuichiro Morishita

**Affiliations:** ^1^Department of Orthopedic Surgery, Spinal Injuries Center, Iizuka, Japan; ^2^Department of Orthopedic Surgery, Kyushuu University, Fukuoka, Japan

## Abstract

**Objective:**

We report two cases of iatrogenic deterioration of lumbar foraminal disc herniations following lumbar disc injections. *Summary of Background Data*. Complications associated with discography were reported. However, only a few reports have thus far referred to the iatrogenic deterioration of lumbar foraminal disc herniations.

**Cases:**

60-year-old and 74-year-old men were treated with MR images of L4-5 foraminal disc herniations without fragment in the spinal canal. The patients underwent discography and disc block for its diagnosis and treatment. After disc injections, both patients complained of deterioration of L4 radiculopathy.

**Results:**

On disco-CT or reexamined MR images after disc injections, herniated fragment was migrated from neural foramen to cranial central spinal canal with was not shown in previous MR images. The herniated fragments were extirpated by means of osteoplastic laminoplasty or transforaminal lumbar interbody fusion with facetectomy. The herniated fragments were migrated from neural foramen to cranial central spinal canal.

**Conclusions:**

The injection of liquid medicine into the nucleus led the intradiscal pressure increased, and the disc fragment might prolapsed through the raptured region of annulus fibrosus and migrated to cranial central spinal canal by anatomical reason. The disc injection may have a risk for deterioration of foraminal disc herniation. Our report is instructive for the management of discography for the diagnosis of foraminal disc herniations.

## 1. Introduction

Incidence of lateral disc herniation has been reported to account for 7% to 12% of all lumbosacral disc herniations [1–3]. Magnetic resonance (MR) image has become essential as the gold standard for diagnosing lumbar disc herniation. Although parasagittal MR images or MR-myelo images give us substantial information about neural foramen, discography-enhanced computed tomography (disco-CT) provides much more clear information of neural foraminal pathology.

Complications associated with discography were reported [4–10]. However, only a few reports have thus far referred to the iatrogenic deterioration of lumbar foraminal disc herniations. We hereby report two cases of iatrogenic deterioration of lumbar foraminal disc herniations following lumbar disc injections.

## 2. Case Presentation

Institutional review board approval was granted, and informed consent was obtained from two patients.

### 2.1. Case 1

**Figure 1:**
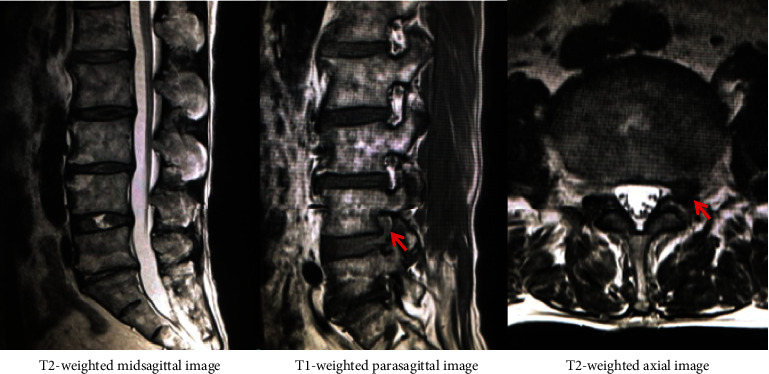
MR images revealed left L4-5 foraminal herniated disc without fragment in the spinal canal.

A 60-year-old man presented with sudden left leg pain in L4 nerve root area. The patient demonstrated neurogenic paralysis on left quadriceps femoris muscle with manual muscle testing 3, hyporeflexia on left patella tendon, and positive left straight leg raising test. MR images revealed left L4-5 foraminal herniated disc without fragment in the spinal canal (Figure 1). Left L4 selective nerve root block remarkably affect. According to the above results, the patients was diagnosed as left L4 nerve root entrapment by left L4-5 foraminal disc herniation.

**Figure 2:**
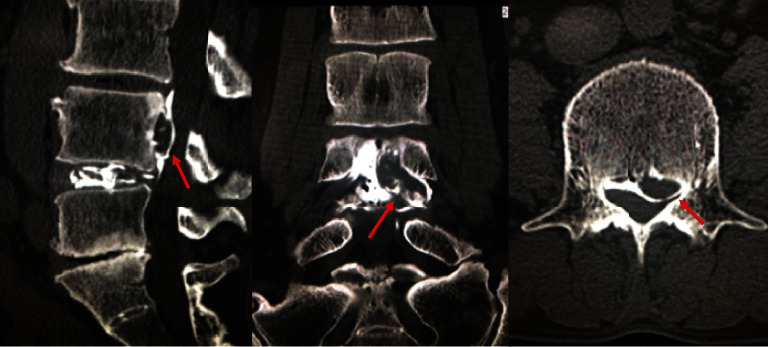
The herniated fragment was migrated from neural foramen to cranial central canal on discography-enhanced CT.

**Figure 3:**
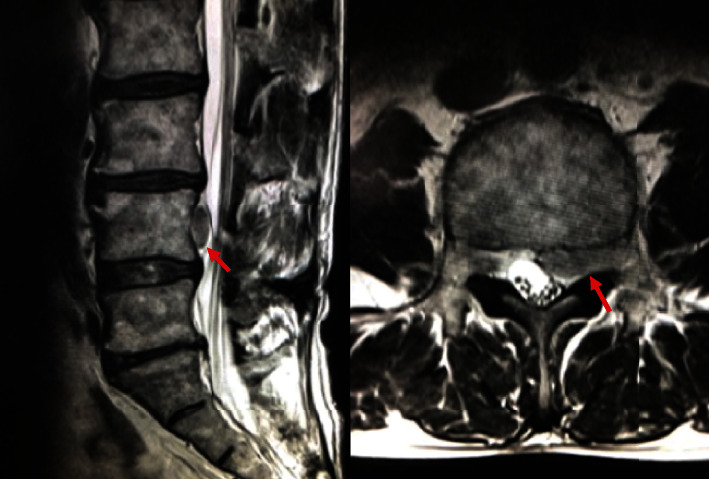
Postdiscogram MR images showed herniated fragment migrated to cranial central canal which was not detected in previous images.

The patient underwent discography for the presurgical imaging diagnosis. The double-needle was inserted to the L4-5 disc, and tip of the needle was located at middle of the nucleus pulposus. Finally, 3.0 cc of Iohexol was injected cautiously without reproduction of left L4 radicular pain. After discography, the patient complained deterioration of left leg numbness without neurological deficit. On disco-CT and postdiscogram MR images, herniated fragment was migrated from neural foramen to cranial central canal which was not detected in previous images (Figures 2 and 3).

**Figure 4:**
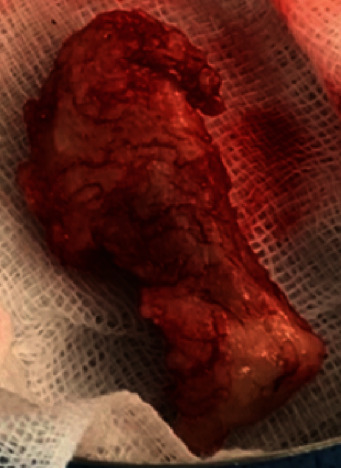
2.5 g-weighted disc fragment with annulus fibrosus.

Herniotomy was performed by osteoplastic approach [11, 12]. Observing the neural foramen, the herniated disc fragment was migrated from neural foramen to cranial central canal. 2.5 g-weighted disc fragment with annulus fibrosus was extirpated with one piece (Figure 4). After surgery, neurological status was fully recovered immediately.

### 2.2. Case 2

**Figure 5:**
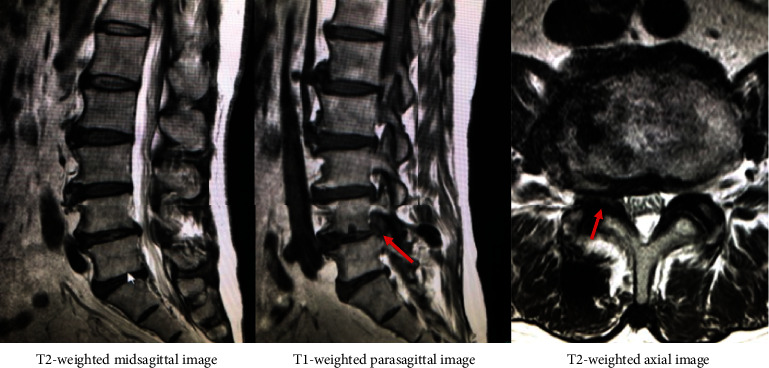
MR images revealed right L4-5 foraminal herniated disc without fragment in the spinal canal.

A 74-year-old man presented with right leg pain in L4 nerve root area. He was treated in previous clinic with diagnose of right L4-5 foraminal disc herniation (Figure 5). After disc block with 1.9 mg of Dexamethasone sodium phosphate and 1.5 ml of mepivacaine hydrochloride, the patients complained of deterioration of right leg pain.

**Figure 6:**
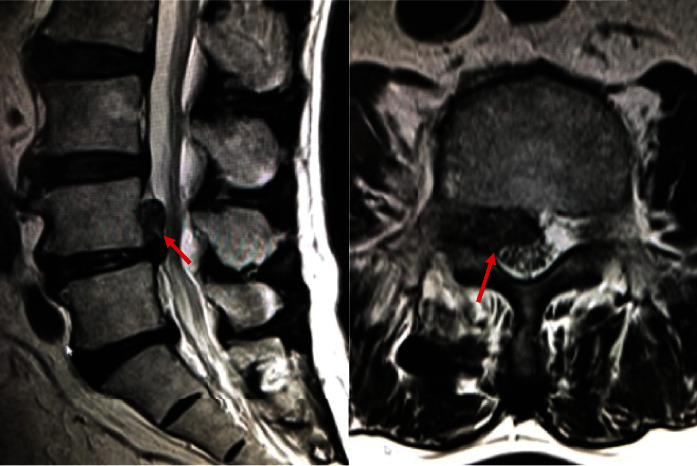
Postdiscogram MR images showed herniated fragment migrated from neural foramen to cranial central canal which was not detected in previous images.

The patient was introduced to our facility, and reexamined MR images showed herniated fragment migrated from neural foramen to cranial central canal which was not detected in previous images (Figure 6). No neurological deficit was observed. Transforaminal lumbar interbody fusion with right L4-5 facetectomy was performed to extirpate the herniated fragment.

## 3. Discussion

Grubb et al. [10] reported only one incidence of disc herniation after injection among 346 injected discs. In our cases, two patients with lumbar foraminal disc herniation failed to have an iatrogenic deterioration of lumbar foraminal disc herniations after disc injection. The injection of liquid medicine into the nucleus led the intradiscal pressure increased and might prolapsed the disc fragment through the raptured region of annulus fibrosus from neural foramen to cranial central canal.

The lumbar posterior longitudinal ligament (PLL) attaches to the posterior aspect of the intervertebral discs and to the adjacent margins of the vertebral bodies [13]. Due to its wide attachment to inferior of the intervertebral disc at the intervertebral foramina, structure presents a denticulate appearance over each vertebral body [14, 15]. Moreover, there is the ligamentum complex in transforaminal and intraforaminal region [16, 17]. Therefore, we hypothesized that the disc fragment might prolapsed through the raptured region of annulus fibrosus where the PLL was defected and migrated not to lateral foramen but to cranial central canal by anatomical reason.

Guyer and Ohnmeiss [18] reviewed the literature dealing with lumbar discography. They concluded that lumbar discography should be performed by those well experienced with the procedure and in sterile conditions with a double-needle technique and fluoroscopic imaging for proper needle placement. Information assessed and recorded should include the volume of contrast injected, pain response, with particular emphasis on its locations and similarity to clinical symptoms, and the pattern of dye distribution. In case 1, careful attention was paid to interpreting the pain response during the injection. However, no pain response was observed during the injection, and totally, 3.0 cc of contrast medium was injected. We reflect on ourselves that 3.0 cc of injection into the nucleus pulposus was overdose even if there was no reproductive pain response. The disc injection may have a risk for deterioration of foraminal disc herniation. Our report is instructive for the management of discography for the diagnosis of foraminal disc herniations.
